# Bilateral Pulmonary Langerhans's Cell Histiocytosis is Surgical Challenge in Children: A Case Report

**DOI:** 10.1055/s-0039-1688771

**Published:** 2019-05-23

**Authors:** Tutku Soyer, Gül Özyüksel, Özlem Boybeyi Türer, Kübra Çakmakkaya, Sinan Yavuz, Bilgehan Yalçın, Diclehan Orhan, Ebru Yalçın, Deniz Doğru, Benan Bayrakçı, Nural Kiper, Canan Akyüz

**Affiliations:** 1 Department of Pediatric Surgery, Hacettepe University, Faculty of Medicine, Ankara, Turkey; 2 Department of Pediatrics, Hacettepe University, Faculty of Medicine, Ankara, Turkey; 3 Department of Pediatric Intensive Care, Hacettepe University, Faculty of Medicine, Ankara, Turkey; 4 Department of Pediatric Oncology, Hacettepe University, Faculty of Medicine, Ankara, Turkey; 5 Department of Pediatric Pathology, Hacettepe University, Faculty of Medicine, Ankara, Turkey; 6 Department of Pediatric Pulmonology, Hacettepe University, Faculty of Medicine, Ankara, Turkey

**Keywords:** pulmonary Langerhans's cell histiocytosis, children, bilateral

## Abstract

**Background** Pulmonary Langerhans's cell histiocytosis (PLCH) is a rare cause of interstitial lung disease in children and more than half of the cases are bilateral. Persistent respiratory distress due to spontaneous pneumothorax (SP) in bilateral PLCH may refractory to conservative treatment and posed a great challenge to surgical modalities. A 3-year-old boy with SP due to bilateral PLCH is presented to discuss the surgical options of recurrent and refractory PLCH cases in children.

**Case Report** The patient was admitted to the emergency department with severe respiratory distress and SP. After chest tube insertion, biopsy from neck mass revealed Langerhans's cell histiocytosis. Chemotherapy including vinblastine and prednisone was initiated. Due to persistent respiratory difficulty and air leaks, talc pleurodesis and thoracoscopic bullae excision with pleural decortication were performed. Two months after the admission, due to nosocomial infection and severe respiratory distress, extracorporeal membranous oxygenation (ECMO) support was initiated. The patient was died of ECMO complications on 24th day of ECMO.

**Conclusion** Despite the use of chemotherapy and surgical excision of cystic lesions, bilateral PLCH in children may have lethal outcome. Other treatment options including respiratory support with ECMO and lung transplantation should be considered as last resort of treatment alternative in persistent cases.

## Introduction

Langerhans's cell histiocytosis (LCH) is a rare disease characterized by abnormal infiltration of certain organs by dendritic cells. 1 Pulmonary involvement in LCH (PLCH) is more common in adults and mostly occurs in smokers. 2 The estimated incidence of LCH is 4 to 9 cases per 1 million in children. 3 4 Pulmonary involvement as an isolated entity is extremely rare in children and usually can be seen as part of a systemic disease. Pathogenesis of PLCH differs from that of adults where most of the affected adult cases are cigarette smokers. However, no causative relationship has been proven between smoking and pediatric PLCH, passive smoking may considered in the etiology. An uncontrolled immune response to an unknown stimulus or antigen is suggested in the pathogenesis in children. 5

The clinical findings of PLCH are nonproductive cough and dyspnea. Spontaneous pneumothorax (SP) is resulting from a rupture of subpleural bullae and cystic lesions occur in 10 to 20% of cases. 6 Pleurodesis, cysts excision, and partial or total pleurectomy are common surgical options. Extensive parenchymal destruction due to fibrosis may lead to respiratory failure and end-stage pulmonary disease. 7 There is no consensus on the optimum treatment of bilateral PLCH in children.

A 3-year-old boy with SP due to bilateral PLCH is presented to discuss the surgical options of recurrent and refractory PLCH cases in children.

## Case Report

**Fig. 1:**
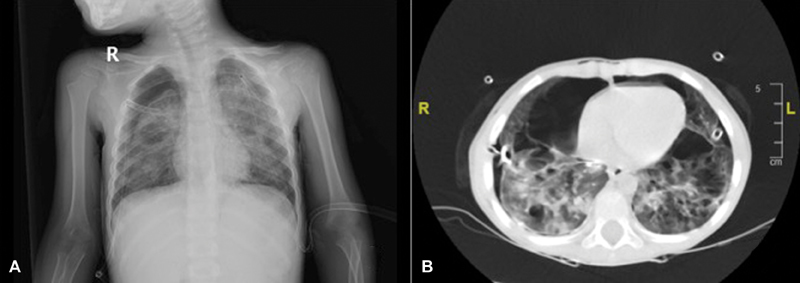
( **A** ) Chest X-ray of the patient showing pneumothorax on right side. ( **B** ) Computed tomography revealing bilateral cysts and ground glass appearance.

**Fig. 2:**
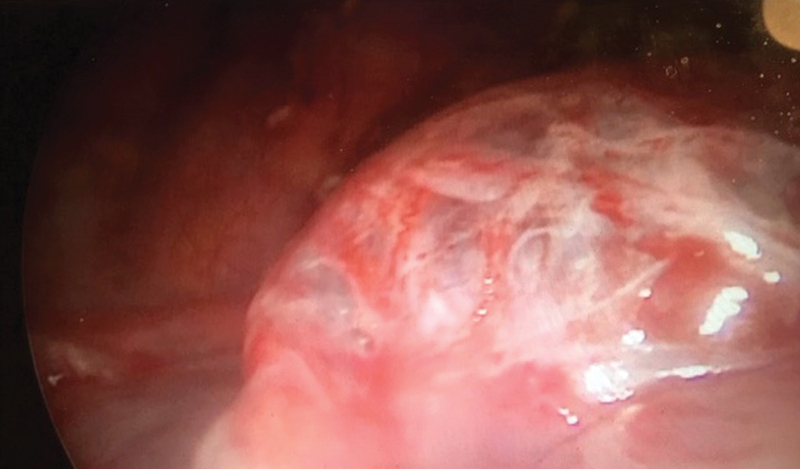
Thoracoscopic image of cystic lesion.

A 3-year-old boy with severe respiratory distress admitted to emergency department. He had pneumothorax at right chest and underwent tube thoracostomy ( Fig. 1A ). In his past medical history, he has free of symptoms and had no chronic disease. None of the family members had lung disease and none of them were smokers. At admission, his vital signs were within normal limits except high respiratory rate (45/minute). Total blood count, liver, and renal function tests were also unremarkable. On physical examination, a 2 × 1 cm palpable mass was noted at right side of neck. Computed tomography (CT) of chest revealed bilateral air cysts in varying sizes with ground glass appearance ( Fig. 1B ). Pneumothorax was also detected. The patient underwent biopsy form the neck mass and diagnosed as LCH confined to right salivary gland. The histopathologic features include polymorph nuclear leucocytes and atypical histocytes with oval nucleus with pale granular cytoplasm. Immunohistochemical staining with CD1a (cluster of differentiation 1 a) and S100 were positive. Chemotherapy including vinblastine and prednisone was initiated. During follow-up, pneumothorax persisted and another chest tube was inserted on the left side. Since the patient was unresponsive to medical treatment 2 weeks after admission, chemical pleurodesis with talc (2 cc) was performed. The pleurodesis was performed through tube thoracostomy. No intrapleural analgesics were used. The tube was clamped for 1 hour and unclamped at the end of procedure. During the clamped period, patients recommended to rotate on each side for 10 minutes. At the end of 10 days follow-up, patient developed bilateral pneumothorax with subcutaneous emphysema despite tube thoracostomy. Thoracoscopic bullae excision with pleural decortication was performed 10 days after talc pleurodesis ( Fig. 2 ). Histopathological evaluation of pleural biopsies obtained by thoracoscopy revealed inflammation and foreign bodies (talc). No lung biopsy was sampled during bullae excision. After surgical excision of subpleural bullae, the patient was symptom free for only 1 week and after developed respiratory insufficiency. Although total pleurectomy was considered for the patient, due to nosocomial pulmonary infection and severe respiratory distress, mechanical ventilation was initiated. Despite high-frequency ventilation, the oxygenation was inadequate and higher pressures are required. Five weeks after the bullae excision, extracorporeal membranous oxygenation (ECMO) support is initiated with vein-venous catheterization via internal jugular vein. At the 24th day of ECMO, he developed cranial bleeding and ventricular herniation and died of bleeding complications.

## Discussion

Langerhans's cell histiocitosis (LCH) is a rare disease occurring in 4 to 9 cases per 1 million children. 8 It encopresis a heterogeneous spectrum of clinical findings and may involve either a single organ or present as multisystem disease. Pulmonary involvement is seen in 25% of all pediatric cases and usually as part of a multiorgan LCH. Isolated PLCH is reported only 1% of pediatric cases. 9 Bilateral PLCH is also extremely rare in children and may have an unpredictable course and lethal outcome. It predominantly affects young adults between the ages 20 and 40 years, and there is no gender predilection. Pediatric PLCH peaks between 1 and 3 years of age. The exact incidence of PLCH in children is unknown because significant numbers of cases are asymptomatic and may resolve spontaneously. 10 Although it constitutes 4 to 5% of all pediatric lung biopsies, histopathologic diagnosis of PLCH is difficult in advanced disease.

The pathogenesis of PLCH in adults is attributed to cigarette smoking and 95% of affected adults are smokers. In children, immune response to an unknown antigen and/or stimulus is suggested as the underlying cause of LCH. PLCH presents with tachypnea, chest pain, dyspnea, cyanosis, chronic, and persistent cough. SP resulting rupture from cystic lesions may occur as initial presentation. Our patient was also admitted to emergency department with SP and dyspnea. The diagnosis of PLCH is characteristically based on the radiologic findings. It may consist of cystic lesions varying in size and frequently involves lower lobes in children. CT typically shows nodular and reticular lesions in early stages of the disease and cystic lesions in advanced disease. CT scan of our patient also suggested advanced PLCH showing cystic lesions with ground glass appearance. The ground glass opacities are very rare radiologic findings of PLCH and correspond to desquamated interstitial pneumonia like changes. 11 According to the stage of disease, different radiologic findings can be seen. Nodular and ground glass appearance also exhibit regression of PLCH, while emphysema and fibrotic changes suggest persistence and progression. Other nodular and interstitial lung diseases should be differentiated from PLCH. The silicosis and sarcoidosis may have similar nodular lesions in chest scans but very rare in children. Also, different from the infectious causes, PLCH has no lymph nodes in perihilar areas.

Children with progressive PLCH can be treated with chemotherapy including vinblastine, prednisone, methotrexate, cyclophosphamide, and etoposide. 12 The clinical response to chemotherapy is unpredictable in children and requires long-term follow-up. Vinblastine and prednisone was the choice of chemotherapy in our patient and partial response was obtained during the medical treatment. The role of passive cigarette smoking in the development of PLCH is controversial in children. However, there is no prospective data suggesting improvement of outcome by stopping smoke exposure; it is highly recommended.

The treatment option of recurrent pneumothoraces is tube thoracotomy in most cases. Other surgical interventions are needed when there is no improvement in respiratory functions after 12-week course of chemotherapy; persistent pneumothorax due to chest tubes or worsening of CT scan findings during follow-up. 13 The conservative treatment of recurrent pneumothoraces in PLCH usually fails since the underlying lungs are very fragile and tend to collapse easily. The recurrence rate of pneumothorax treated with chest tube is only 58%. 14 Chemical pleurodesis has limited rate of success in the treatment of extensive PLCH and not recommended if there is a possibility of lung transplantation. At the early stages of disease, we used chemical pleurodesis with talc after full extension of lungs with tube thoracostomy. However, talc pleurodesis was failed and did not prevent recurrent pneumothoraces. Abdul Aziz et al reported that mechanical pleurodesis is superior to chemical pleurodesis. 13 It can be performed by either with thoracotomy or thoracoscopy. It is not considered as a contraindication for transplantation. Therefore, thoracscospic excision of cysts with partial pleurectomy was performed. In extensive PLCH, partial pleurectomy and/or abrasion of pleura may not be sufficient to eradicate persistent pneumothorax. Total pleurectomy is an aggressive surgical alternative that can be used in recurrent pneumothorax. Although better outcomes are reported, it should be the last resort of surgical treatment in children. For our patient, authors discussed to perform bilateral total pleurectomy as last surgical option to avoid recurrence. But, due to nosocomial respiratory infection and need of ventilator support, it was not possible to perform total pleurectomy.

Lung transplantation is a therapeutic option in selected cases with progressive disease. 7 It should be considered when severe pulmonary hypertension exists and not response to vasodilator treatment. Surgical pleurodesis did not preclude lung transplantation. ECMO has been shown to be a valid alternative as a bridge for lung transplantation in PLCH. 15 It was not considered as an alternative treatment in our case because of severe deterioration of the patient.

The prognosis of PLCH is variable and unpredictable in pediatric age group. The poor outcome of the PLCH is related with extremes age, prolonged worse conditions, multiorgan involvement, reduced diffusion capacity, extensive cysts and honeycombing on imaging, recurrent pneumothoraces, pulmonary hypertension, and prolonged treatment. 16 Death may occur due to pulmonary hypertension and/or severe respiratory insufficiency. Our patient had most of the poor prognostic factors and died due to severe pulmonary insufficiency and ECMO complications.

## Conclusion

In conclusion, despite the use of chemotherapy and surgical excision of cystic lesions, bilateral PLCH in children may have lethal outcome. Other treatment options including respiratory support with ECMO and lung transplantation should be kept in mind in persistent cases.

## Notes

New Insights and the Importance for the Pediatric Surgeon Despite the use of chemotherapy and surgical excision of cystic lesions, bilateral pulmonary Langerhans's cell histiocytosis in children may have lethal outcome. Other treatment options including respiratory support with extracorporeal membranous oxygenation and lung transplantation should be considered in persistent cases.
